# Insomnia and Its Impact on Psychomotor Reactivity, Autonomic Function, and Psychological Well-Being Among Medical Students: A Cross-Sectional Analytical Study

**DOI:** 10.7759/cureus.96619

**Published:** 2025-11-11

**Authors:** Balasubramaniam Dhanusri, Rajasegaran Rajalakshmi, Praveen Prakash, Balaji Bharadwaj, Harichandrakumar KT

**Affiliations:** 1 Department of Physiology, Jawaharlal Institute of Postgraduate Medical Education and Research, Puducherry, IND; 2 Department of Psychiatry, Jawaharlal Institute of Postgraduate Medical Education and Research, Puducherry, IND; 3 Department of Biostatistics, Jawaharlal Institute of Postgraduate Medical Education and Research, Puducherry, IND

**Keywords:** auditory reaction time, body composition, body composition, heart rate variability (hrv), insomnia disorder, insomnia severity index, medical school students, visual reaction time

## Abstract

Background

Insomnia is a common sleep disorder associated with impaired cognitive function, psychological distress, and adverse health outcomes. Medical students, owing to their demanding academic schedules and lifestyle patterns, are particularly vulnerable to sleep disturbances. This study aimed to determine the prevalence and severity of insomnia among medical students and to explore its associations with cardiac autonomic status, psychomotor vigilance, psychological well-being, and body composition.

Methods

In this cross-sectional analytical study, a total of 118 second-, third-, and final-year MBBS students were recruited using stratified cluster random sampling. Insomnia severity was assessed using the Insomnia Severity Index (ISI). Psychological well-being was evaluated with the Depression, Anxiety, and Stress Scale (DASS-21). Auditory and visual reaction times (RTs) were measured using the RTM-608 Response Analyzer. Short-term heart rate variability (HRV) was analyzed using a 5-minute ECG recording. Anthropometric measures and body composition were assessed using standardized protocols and bioelectrical impedance analysis. Data were analyzed using appropriate parametric and non-parametric tests.

Results

The prevalence of clinically significant insomnia (ISI ≥10) was 27.1% (95% CI: 19.9%-35.8%). According to ISI classification, 58.5% of participants had no insomnia, 37.3% had subthreshold insomnia, 3.4% had moderate clinical insomnia, and 0.8% had severe insomnia. Compared to participants without insomnia, those with subthreshold insomnia had significantly longer auditory (p < 0.001) and visual RTs (p = 0.002), and higher DASS-21 scores (p < 0.001), but no significant differences in HRV indices or body composition. Correlation analysis showed that ISI scores were positively associated with auditory RT (r = 0.456, p < 0.001), visual RT (r = 0.247, p = 0.007), and DASS-21 scores (r = 0.508, p < 0.001).

Conclusions

Insomnia was highly prevalent among medical students and was significantly associated with poorer psychomotor performance and greater psychological distress, though not with HRV or body composition. These findings suggest that the cognitive and psychological consequences of insomnia may manifest earlier than autonomic or metabolic alterations in young adults. Early screening and targeted interventions are warranted to promote sleep health in this vulnerable population.

## Introduction

Insomnia, or insomnia disorder, is a sleep disorder characterized by inadequate sleep quantity, sleep quality, or both [1]. The third edition of the International Classification of Sleep Disorders (ICSD-3) [2] defines insomnia as “a persistent difficulty with sleep initiation, duration, or consolidation that occurs despite adequate opportunity and circumstances for sleep and results in concern, dissatisfaction, or perceived daytime impairment, such as fatigue, decreased mood or irritability, general malaise, or cognitive impairment.”

A recent systematic review reported the global prevalence of insomnia at 16.2% [3]. In the Indian context, estimates for the prevalence of insomnia stood at 25.7% [4], and among South Asian university students in general, prevalence figures were as high as 49% [5]. Medical students, a unique subset of young adults, face distinct challenges that heighten their vulnerability to sleep disturbances. A systematic review conducted to assess insomnia among university students in general showed a weighted mean prevalence of 18.5% [6], whereas a meta-analysis conducted on medical students in particular reported a pooled prevalence of 55.64% [7], approximately three times higher than the former. Their demanding academic curriculum, clinical responsibilities, and personal commitments, along with lifestyle factors such as excessive screen exposure, prolonged study hours, irregular sleep patterns, and circadian misalignment, make them particularly susceptible to the detrimental effects of inadequate sleep [8].

Inadequate sleep, in both quantity and quality, is associated with wide-ranging adverse outcomes across physical, psychological, and social health domains. A reduction in sleep duration has been linked to adverse effects on the nervous, cardiovascular, endocrine, respiratory, and immune systems, and is an established risk factor for several chronic ailments, including diabetes, hypertension, and cardiovascular and cerebrovascular disorders [9]. In the psychological domain, sleep impairment and insomnia have been linked to aberrant behavior, decreased psychomotor vigilance, impaired decision-making, and increased lapses in attention [9]. It is also associated with increased irritability, reduced tolerance to frustration, and mood disturbances, which can result in impaired social functioning [9].

This study aimed to determine the prevalence and severity of insomnia among medical students and its associations with cardiac autonomic status, body composition, and auditory and visual reaction times (RTs).

## Materials and methods

This was a cross-sectional analytical study conducted from May to August 2025 in the Department of Physiology at the Jawaharlal Institute of Postgraduate Medical Education and Research (JIPMER), Puducherry, India. The study was approved by the Institute Research Monitoring Committee and the Institute Ethics Committee (JIP/IEC-0S/2024/598).

Objectives

The primary objective of the study was to determine the prevalence and severity of insomnia among medical students using the Insomnia Severity Index (ISI) [10]. The secondary objectives were to assess cardiac autonomic status through heart rate variability (HRV), reaction time (RT) using auditory and visual reaction time (VRT) tests, body composition using bioelectrical impedance analysis (BIA), and psychological well-being using the Depression, Anxiety, and Stress Scale (DASS-21) [11], as well as to examine the associations of these parameters with insomnia severity.

Sample size estimation and sampling

The required sample size was estimated using the single-proportion formula, based on a systematic review reporting the prevalence of insomnia among South Asian university students to range between 35.4% and 70% [5]. A conservative prevalence of 50% was assumed, with a 5% level of significance and an absolute precision of 7%, yielding a calculated sample size of 196 participants.

A stratified cluster random sampling technique was used to ensure both representativeness and feasibility. The study population comprised second-, third-, and final-year MBBS students, with approximately 180 students in each academic year. For academic purposes, each year was divided into four batches of 40-45 students. To maintain randomness and proportional representation, two batches from each academic year (a total of six batches) were randomly selected using OpenEpi software [12].

Although the required sample size was 196, the final number of participants recruited was 118, reflecting a response rate of approximately 60%. Informed written consent was obtained from each participant prior to enrollment.

Inclusion and exclusion criteria

Participants were apparently healthy adults aged 19-25 years, of both genders, and enrolled in the second, third, or final year of the MBBS program at the Institute. Individuals with a documented history of chronic or acute medical illness, major sleep disorders such as sleep apnea or narcolepsy, or psychiatric disorders including intellectual disability or organic brain syndromes were excluded. Participants with substance dependence or those receiving medications known to affect sleep, such as stimulants, antidepressants, decongestants, or beta-blockers, were also excluded. Additional exclusion criteria included engagement in rotating shift work, recent or ongoing jet lag, or exposure to significant emotional stressors such as bereavement, family conflict, or traumatic events. Students undergoing major academic assessments, including internal examinations or viva voce, or participating in cultural events within two weeks prior to recruitment or in the week following enrollment, were likewise deemed ineligible.

Assessment of insomnia and perceived severity

For the detection of insomnia and assessment of its severity, the ISI questionnaire was used, which evaluates the presence and perceived severity of insomnia symptoms [10]. A license for its use was obtained prior to the start of data collection. The ISI is a validated, self-administered, 7-item Likert-type scale, with each item scored from 0 to 4. The total score ranges from 0 to 28 and is categorized as follows: 0-7, no clinically significant insomnia; 8-14, subthreshold insomnia; 15-21, clinical insomnia of moderate severity; and 22-28, clinical insomnia of severe intensity. A score of 10 is recommended as the cutoff point when the ISI is employed for diagnostic purposes [13]. The questionnaire was administered to all study participants.

Assessment of psychological well-being

Depression, anxiety, and stress levels perceived by the subjects over the past week were assessed using the self-rated 21-item Depression Anxiety Stress Scale (DASS-21) questionnaire [11], which is in the public domain. The tool includes three subscales to assess depression, anxiety, and stress. The study participants were asked to rate their experience of each symptom using a 4-point Likert scale. Based on standard scoring guidelines, the subscale scores were summed and categorized into mild, moderate, severe, and extremely severe levels.

Cognitive assessment: auditory and VRT

RT, a measure of sensorimotor performance and alertness [14], was assessed for both auditory and visual stimuli using the RTM-608 Response Analyzer (Medicaid Systems, Chandigarh, India), which has a clock resolution of 0.1 milliseconds.

Auditory reaction time (ART) was defined as the time taken to respond to auditory stimuli of two frequencies: high-frequency (HF; ~1 kHz) and low-frequency (~500 Hz). VRT was defined as the time taken to respond to color stimuli, specifically red and green lights.

Participants were seated comfortably on a stool and provided with sufficient practice trials to familiarize themselves with the procedure. They were instructed to press the response switch using the index finger of their dominant hand as soon as the stimulus was perceived. The corresponding RT, measured in milliseconds, was then displayed on the instrument. For each modality, three readings were recorded with a randomized interval of 2-5 seconds between stimuli to minimize anticipatory responses, and the mean value was taken as the participant’s RT.

Assessment of short-term HRV

Cardiac autonomic status was assessed using the short-term heart rate variability (s-HRV) analysis technique. A Lead II ECG was recorded for 5 minutes after the participant had rested in a supine position for 15 minutes, utilizing the BIOPAC MP150 system. For s-HRV analysis, R-R intervals were derived from the 5-minute ECG recording after filtering out ectopic beats and noise. These R-R intervals were then analyzed using Kubios HRV version 2.0 software (Kubios Oy, Kuopio, Finland), adhering to the guidelines established by the Task Force of the European Society of Cardiology and the North American Society of Pacing and Electrophysiology [15]. Both time-domain and frequency-domain HRV indices indicative of sympathetic and parasympathetic activity were analyzed.

Time-domain indices of HRV provide important insights into the autonomic modulation of cardiac function. The SD of all normal-to-normal (NN) R-R intervals reflects overall HRV and represents the combined influences of both sympathetic and parasympathetic activity. Root mean square of successive differences (RMSSD) between adjacent NN intervals is primarily an index of parasympathetic activity, capturing short-term variations in heart rate. Similarly, the proportion of adjacent NN intervals differing by more than 50 ms (pNN50) serves as another marker of parasympathetic modulation, indicating the relative frequency of significant beat-to-beat interval differences throughout the recording period.

Frequency-domain analysis of HRV provides additional information on autonomic modulation by decomposing heart rate fluctuations into their spectral components. In this study, R-R interval segments of 256 seconds were processed through cubic-spline interpolation, detrending, and application of a Hanning window, followed by fast Fourier transformation using Welch’s periodogram averaging method to estimate the power spectral density. Low-frequency (LF) power was obtained by integrating the spectral power within the 0.04-0.15 Hz band, representing a combination of sympathetic and parasympathetic influences. HF power, calculated within the 0.15-0.4 Hz band, primarily reflects parasympathetic activity. To account for variations in total power, LF normalized units (LFnu) and HF normalized units (HFnu) were derived by dividing LF and HF power, respectively, by total power, serving as relative indicators of sympathetic and parasympathetic activity. The LF/HF ratio, computed by dividing LF power by HF power, was used as an index of sympathovagal balance, reflecting the relative contributions of sympathetic and parasympathetic modulation.

Assessment of anthropometric indices and body composition

Anthropometric parameters including height, weight, neck circumference, waist circumference, and hip circumference were measured in accordance with the International Standards for Anthropometric Assessment [16]. Body composition was assessed using the BIA technique with the Bodystat QuadScan 4000 apparatus (Isle of Man, UK). The BIA technique estimates body composition by measuring the electrical impedance of a low-intensity, HF alternating current as it passes through the body.

For the procedure, participants were placed in the supine position, and signal-inducing and sensor electrodes were positioned as per the manufacturer’s recommendations. After entering individual anthropometric details, the analyzer provided estimates of body composition indices, including total body water, body fat (kg), body fat mass index (BFMI), and fat-free mass index (FFMI).

Statistical analysis

Data were collected in a digital spreadsheet and subsequently analyzed using SPSS version 19 (IBM, Armonk, NY, USA). Categorical variables such as gender and insomnia severity category are expressed as frequencies and percentages. Quantitative variables including age, insomnia severity score, HRV indices, RT, body composition parameters, and DASS-21 scores are presented as mean ± standard deviation or median (IQR), according to the results of normality testing.

Bivariate correlation analysis was performed to examine the relationship between insomnia severity and other continuous variables. Pearson’s correlation coefficient was applied to normally distributed variables, while Spearman’s rank correlation was used for non-normally distributed data. For subgroup analysis, participants with no clinically significant insomnia and those with subthreshold insomnia were compared. Prior to analysis, assumptions of normality and homogeneity of variance were verified, and an independent t-test was employed to compare mean values between the two subgroups.

## Results

A total of 118 students participated in the study, comprising 75 males (63.6%) and 43 females (36.4%). The median age was 19 years (interquartile range: 1 year), with ages ranging from 18 to 24 years. The mean BMI of the sample was 22.5 ± 3.4 kg/m² (Table 1).

**Table 1:** Demographic details of the study participants (N = 118).

Parameters	Category (if applicable)	Measures
Age (years), Median (IQR)	-	19 (19-20)
Sex, Frequency (%)	Male	75 (63.6%)
	Female	43 (36.4%)
BMI (kg/m²), Mean ± SD	-	22.5 ± 3.4

According to the ISI classification (Table 2), 69 participants (58.5%) scored below the cut-off of 8 and were categorized as having no clinically significant insomnia. A further 44 participants (37.3%) scored between 8 and 14, consistent with subthreshold insomnia. Four participants (3.4%) met the criteria for clinical insomnia of moderate severity (scores 15-21), while one participant (0.8%) met the criteria for severe clinical insomnia (scores 22-28). When a cut-off score of ≥10 was applied to detect insomnia, 32 participants (27.1%; 95% CI: 19.3%-36.1%) were classified as having insomnia.

**Table 2:** Insomnia status and severity among the study participants. Ordinal classification is based on the Insomnia Severity Index (ISI) scored from 0-28. 0-7: No clinically significant insomnia; 8-14: Subthreshold insomnia; 15-21: Clinical insomnia (moderate severity); 22-28: Clinical insomnia (severe).

Parameter	Frequency (%) (Total N = 118)
Participants without insomnia	69 (58.5%)
Participants with subthreshold insomnia	44 (37.3%)
Participants with clinical insomnia - moderate	4 (3.4%)
Participants with clinical insomnia - severe	1 (0.8%)

The gender distribution across these four groups is presented in Table 3. The male-to-female ratio between the group without insomnia and the group with subthreshold insomnia was comparable (Pearson’s chi-square test, p = 0.84).

**Table 3:** Sex distribution across insomnia severity among the study participants.

Sex	Insomnia Status				Total
	No insomnia (N = 69)	Subthreshold (N = 44)	Moderate (N = 4)	Severe (N = 1)	
Male	43 (62.3%)	29 (65.9%)	3 (75.0%)	0 (0.0%)	75
Female	26 (37.7%)	15 (34.1%)	1 (25.0%)	1 (100.0%)	43

Results of the subgroup analysis comparing participants without insomnia and those with subthreshold insomnia are presented in Table 4. No significant differences were observed between the groups with respect to demographic variables, including age, height, weight, and BMI. Participants with subthreshold insomnia had significantly higher ISI scores (p < 0.001), longer auditory reaction times (p < 0.001), longer visual reaction times (p = 0.002), and higher DASS-21 scores (p < 0.001) compared to those without insomnia. In contrast, heart rate variability indices and body composition parameters did not differ significantly between the groups (Table 4).

**Table 4:** Comparison of parameters between participants with no insomnia and those with subthreshold insomnia. The independent t-test was used to compare means across the two groups. * Considered statistically significant at p < 0.01. ** Considered statistically significant at p < 0.001. ISI: Insomnia Severity Index; DASS-21: 21-item Depression Anxiety Stress Scale; HF: High-frequency band (0.15-0.40 Hz) power spectral density; HR: Heart rate; HRV: Heart rate variability; LF: Low-frequency band (0.04-0.15 Hz) power spectral density; LF/HF ratio: Ratio between low-frequency and high-frequency band power spectral densities; nu: Normalized units (relative power); RMSSD: Root mean square of successive differences between adjacent NN intervals; RR: ECG R-R interval; SDNN: Standard deviation of NN intervals; BFMI: Body fat mass index; FFMI: Fat-free mass index.

Parameter	No insomnia (N = 69)	Subthreshold insomnia (N = 44)	p-value
Age (years)	19.52 ± 0.70	19.77 ± 0.96	0.11
Height (cm)	168.60 ± 10.08	168.88 ± 9.97	0.89
Weight (kg)	64.13 ± 12.97	65.51 ± 12.17	0.57
BMI (kg/m²)	22.39 ± 3.27	22.96 ± 3.64	0.4
Severity of Insomnia			
ISI score	4.07 ± 2.01	10.57 ± 1.98	<0.001**
Psychomotor			
Auditory reaction time (ms)	110.7 ± 5.4	116.7 ± 6.4	<0.001**
Visual reaction time (ms)	118.2 ± 6.5	123.8 ± 11.8	0.002*
Psychological			
DASS-21 score	9.22 ± 7.07	15.30 ± 8.75	<0.001**
Heart Rate Variability			
Mean RR (ms)	839.95 ± 139.61	856.39 ± 162.82	0.57
SDNN (ms)	66.93 ± 44.00	70.24 ± 54.11	0.73
RMSSD (ms)	82.88 ± 66.16	90.30 ± 95.91	0.63
NN50 (count)	128.88 ± 109.09	128.91 ± 90.55	0.99
pNN50 (%)	36.62 ± 24.27	38.94 ± 25.07	0.63
LFnu	42.28 ± 18.92	40.52 ± 14.74	0.6
HFnu	57.40 ± 18.81	59.30 ± 14.67	0.57
LF/HF ratio	1.05 ± 1.12	1.11 ± 1.54	0.83
Body Composition Parameters			
Total body water (kg)	57.32 ± 8.54	57.80 ± 8.62	0.78
Body fat (kg)	10.48 ± 5.09	10.31 ± 6.06	0.88
Lean body mass (kg)	52.68 ± 11.97	55.18 ± 11.51	0.29
BFMI (kg/m²)	3.82 ± 2.04	3.72 ± 2.44	0.82
FFMI (kg/m²)	18.52 ± 2.74	19.08 ± 2.49	0.3

Correlation analysis demonstrated significant moderate positive associations between insomnia severity (ISI score) and measures of psychomotor performance and psychological distress (Table 5). ISI scores showed a moderate positive correlation with auditory reaction time (r = 0.456, p < 0.001) and with DASS-21 scores (r = 0.508, p < 0.001). A weaker but significant positive correlation was also observed between ISI scores and visual reaction time (r = 0.247, p = 0.007). These findings indicate that greater insomnia severity was associated with slower reaction times and higher levels of depression, anxiety, and stress.

**Table 5:** Correlation between severity of insomnia (ISI score), auditory and visual reaction times, DASS-21 score, HRV, and body composition parameters. Pearson’s correlation was used as the statistical test. r represents Pearson’s correlation coefficient. * Considered statistically significant at p < 0.01. ** Considered statistically significant at p < 0.001. ISI: Insomnia Severity Index; DASS-21: 21-item Depression Anxiety Stress Scale; HF: High-frequency band (0.15-0.40 Hz) power spectral density; HR: Heart rate; HRV: Heart rate variability; LF: Low-frequency band (0.04-0.15 Hz) power spectral density; LF/HF ratio: Ratio between low-frequency and high-frequency band power spectral densities; nu: Normalized units (relative power); RMSSD: Root mean square of successive differences between adjacent NN intervals; RR: ECG R-R interval; SDNN: Standard deviation of NN intervals; BFMI: Body fat mass index; FFMI: Fat-free mass index.

Parameter compared to ISI score	Correlation coefficient, r (N = 118)	p-value
Auditory reaction time	0.456	<0.001**
Visual reaction time	0.247	0.007*
DASS-21 score	0.511	<0.001**
Mean RR (ms)	0.102	0.279
SDNN (ms)	0.083	0.38
RMSSD (ms)	0.117	0.214
NN50 (count)	0.096	0.309
pNN50 (%)	0.124	0.189
LFnu	-0.102	0.278
HFnu	0.106	0.262
LF/HF ratio	-0.036	0.705
Total body water (kg)	0.028	0.776
Body fat (kg)	-0.133	0.169
Lean body mass (kg)	0.021	0.827
BFMI (kg/m²)	-0.135	0.162
FFMI (kg/m²)	0.032	0.744

## Discussion

Sleep disturbances, particularly insomnia, are increasingly recognized as a major concern among university students [5, 6]. A systematic review conducted across university students in general showed an estimated weighted mean prevalence of insomnia of 18.5% [6]. A meta-analysis of studies assessing insomnia in medical students, however, reported an estimated pooled prevalence of 55.64% [7]. This disparity suggests that the academic rigor, prolonged study hours, irregular schedules, and psychological stressors inherent to medical training may significantly contribute to the development and persistence of sleep disturbances and insomnia in this population group. Insomnia has been linked to impaired cognitive performance, emotional dysregulation, and adverse health outcomes, making its assessment in this population both timely and important. The present study was undertaken to determine the prevalence and severity of insomnia among medical students and to examine its associations with cardiac autonomic function, psychomotor speed, psychological well-being, and body composition. By focusing on this high-risk group, the study provides insights into how sleep disturbances may influence not only mental health but also cognitive efficiency and physiological regulation in young adults pursuing a demanding professional education.

In this study, it was observed that 41.5% of respondents reported an ISI score of 8 or above, implying, at the very least, that these participants had symptoms corresponding to subthreshold insomnia, in accordance with the grading guidelines suggested by the ISI questionnaire [10]. However, only five participants (4.6%) had scores exceeding 14, implying clinical insomnia, with four participants classified as moderate and one as severe, according to the guidelines suggested by the ISI questionnaire authors. There is a recommendation to use a cutoff score of 10 when the ISI is employed as a diagnostic screening tool [13]. Accordingly, the prevalence of insomnia in our study population was 27.1%, with a 95% CI of 19.3% to 36.1%.

Furthermore, greater insomnia severity was associated with slower auditory and visual RTs, which is consistent with the literature linking insufficient or fragmented sleep to vigilance lapses and psychomotor slowing. Recent reviews highlight that the Psychomotor Vigilance Test (PVT) and similar RT tasks are highly sensitive to sleep loss and insomnia symptoms, showing clear dose-response effects on lapses and median RT [17, 18]. Experimental studies also demonstrate that RT becomes slower after total sleep deprivation, along with brain activity changes that indicate reduced attention (e.g., increased P300 latency) [19]. Together, these findings support the current result that higher ISI scores are linked to slower auditory and visual RT, likely due to reduced alertness and unstable attention when sleep is inadequate.

In our study population, insomnia severity showed a positive correlation with DASS-21 scores. This is consistent with evidence that poor sleep and insomnia symptoms are closely linked to higher levels of stress, anxiety, and depression among university students. A large meta-analysis conducted on studies involving university students reported consistent associations between impaired sleep and stress [20], and a systematic review similarly documented strong associations between insomnia symptoms and psychological distress in undergraduates [21]. Recent multinational studies in medical students also show parallel patterns, namely poorer sleep quality accompanying worse DASS-21 scores [22]. These convergent findings reinforce the bidirectional relationship between disturbed sleep and mood/arousal dysregulation. This relationship can be explained by the reciprocal influence exerted by sleep and emotional regulatory systems on each other, exemplified by the fact that nearly all mood disorders exhibit sleep disturbances in their symptomatology [23]. The rapid eye movement (REM) stage of sleep, based on neurotransmitter activity, has been shown to be the stage during which the processing of emotional memories occurs, leading to more adaptive responses to stress-inducing experiences [23]. Reduced or fragmented sleep can impair this process, predisposing individuals to greater emotional lability and negative mood [22, 23]. Conversely, elevated stress and anxiety activate physiological arousal pathways that interfere with sleep continuity [23]. Thus, the direction of causality is bidirectional; however, the exclusion of participants with major stressors or psychiatric disorders in this study suggests that poor sleep itself may have contributed to higher DASS-21 scores in this non-clinical sample.

In contrast to RT and DASS-21, HRV indices did not differ significantly between the subthreshold-insomnia group and the group without insomnia. The broader literature on insomnia and HRV remains mixed. A 2023 meta-analysis found that the evidence does not consistently support reduced HRV in individuals with insomnia disorder, reporting only small overall effects and considerable uncertainty [24]. By comparison, a 2025 systematic review and meta-analysis of sleep deprivation experiments found meaningful autonomic shifts (reduced vagal indices) under acute sleep loss [25]. Meanwhile, recent large-scale analyses of comorbid insomnia and obstructive sleep apnea (COMISA) show clearer nocturnal autonomic alterations than in isolated insomnia [26]. Taken together, HRV changes may be minimal in community samples with milder insomnia and more apparent under acute sleep deprivation or comorbid conditions, which is consistent with the lack of differences we observed in our group comparison.

Body composition measures did not differ significantly across insomnia strata in our sample. At the population level, evidence on insomnia and adiposity is heterogeneous. A systematic review suggests that sleep disturbances are associated with higher odds of being overweight [27], and a meta-analysis reports increased obesity risk among people with insomnia symptoms [28]. However, longitudinal work indicates that person-level trajectories of BMI and insomnia can be decoupled or only weakly coupled depending on life stage and covariates [29]. There are also recent data from university staff showing an inverse association between poor sleep quality and BMI after adjustment [30]. Therefore, in young, relatively lean student samples, body composition contrasts between normal and insomnia groups are expected to be small, aligning with the present findings.

Strengths

This study has several strengths. It employed a validated set of tools, including the ISI, DASS-21, HRV, BIA, and standardized reaction-time measures, ensuring methodological rigor and comparability with previous research. The use of stratified cluster random sampling enhanced representativeness across different academic years and reduced the likelihood of selection bias. The study also provided a comprehensive assessment of insomnia by concurrently evaluating its cognitive, psychological, autonomic, and body-composition correlates within a single cohort of medical students.

Limitations

A limitation of this study is that the achieved sample size (n = 118) was lower than the estimated requirement (n = 196). This shortfall may have reduced the statistical power and increased the margin of error, potentially affecting the generalizability of the findings. A post-hoc precision analysis showed that with 118 participants, the 95% confidence interval around an assumed prevalence of 50% was 40.7%-59.3%, corresponding to an absolute precision of ±9.0%, instead of the initially intended ±7%. Nevertheless, the use of stratified cluster random sampling ensured adequate representation across academic years, thereby strengthening the internal validity of the study.

In addition, reliance on the ISI and the DASS-21 scale, though validated and widely used, precluded a full clinical evaluation. This limited the ability to distinguish between primary and secondary insomnia and to clinically confirm psychological well-being.

## Conclusions

This study highlights the significant impact of insomnia among medical students and its clear links with psychomotor performance and psychological well-being. Higher insomnia severity was associated with slower reaction times and increased stress, anxiety, and depression, whereas no meaningful differences were observed in heart rate variability or body composition across insomnia categories. These results suggest that, in young adults, the early effects of sleep problems may be more apparent in cognitive and psychological domains than in autonomic or metabolic measures.

Given the high rates of insomnia symptoms in this group, early detection and targeted interventions to improve sleep habits and psychological resilience are essential to prevent long-term health problems. To address this, colleges could implement routine sleep and mental health screenings, along with workshops on sleep hygiene and time management strategies. Incorporating sleep education and counseling programs into the medical curriculum is also a promising approach.

From a clinical perspective, institutions can ensure access to evidence-based interventions such as cognitive behavioural therapy for insomnia (CBT-I) and mindfulness training. Addressing underlying stressors is equally important for prevention and effective management. Active measures can include evaluating the intensity and structure of academic schedules and introducing flexibility where feasible. Peer support programs may also help reduce stress and promote healthy sleep habits, as students can support each other in staying accountable and motivated.

Finally, broader public health initiatives that raise awareness about sleep hygiene and the role of sleep in mental health can help curb the poor habits that lead to chronic sleep problems. Overall, interventions that promote a cultural shift towards positive sleep hygiene practices may be particularly effective among university students.
